# Melodic and articulatory development is delayed in deaf infants aged 2–4 months

**DOI:** 10.1038/s41598-025-16820-w

**Published:** 2025-08-26

**Authors:** Kathleen Wermke, Sarah Arnold, Wafaa Shehata-Dieler, Mario Cebulla, Johannes Wirbelauer, Philip J. Schluter

**Affiliations:** 1 Center for Pre-Speech Development & Developmental Disorders, University Hospital, University Wuerzburg, Pleicherwall 2, 97070 Würzburg, Germany; 2 Department of Otorhinolaryngology, Head and Neck Surgery, University Hospital, University Wuerzburg, Würzburg, Germany; 3 Children’s Hospital, University Hospital, University Wuerzburg, Würzburg, Germany; 4 School of Health Sciences, University of Canterbury - Te Whare Wānanga o Waitaha, Christchurch, New Zealand; 5 School of Clinical Medicine, Primary Care Clinical Unit, The University of Queensland, Brisbane, Australia

**Keywords:** Infant, Hearing, Cooing, Vocal development, Developmental biology, Psychology, Health care, Medical research

## Abstract

There is a paucity of knowledge about the early vocal development of infants with profound sensorineural hearing loss. In a search for potential differences in cooing vocalisations between hearing impaired (*n* = 12) and healthy infants (*n* = 12) aged 2–4 months, more than 2300 sounds were quantitatively analysed. Using objective criteria, laryngeal activity was analysed. Vocalisation melodies (time function of fundamental frequency) were recorded and grouped into simple (single arc) or complex (multiple arc) melody pattern. Occurrence of supra-laryngeal (articulatory) activity was also analysed using frequency spectrograms. Statistical analysis employed multilevel mixed-effects modified Poisson regression models, with cooing vocalisations nested within infants. Infant age, but not sex, was significantly associated with increasing complexity in both vocal activities over the observation period. Constrained auditory feedback affects both laryngeal and articulatory activity. The group of hearing impaired infants produced significantly less melodic complexity and their cooing showed fewer articulatory activity. Recognition of a delay in these early developmental processes will considerably improve our understanding of deviations in early preparatory processes for language acquisition in hearing impaired infants. Most importantly, it highlights the need to address the lack of objective, detailed studies of pre-lingual sound production in the context of increasingly earlier hearing aid provision.

**Supplementary Information:**

The online version contains supplementary material available at 10.1038/s41598-025-16820-w.

## Introduction

Early identification and treatment of hearing impairment in infants is of paramount importance for the development of language, communication and social skills. The prevalence of hearing loss of 40dB or more is estimated to be 1.86 per 1000 live births in high-income countries^1^. Although this estimated prevalence varies between regions. In the United States, approximately 2–3 infants from every 1,000 live births are born with a detectable level of hearing loss in one or both ears^2^. While, in Germany, approximately 1–3 per 1000 infants are affected by hearing impairment, with around 1.3 per 1000 newborns diagnosed at birth with congenital bilateral hearing disorders^3^. However, the robustness of these rates is not without question^4^.

Infants’ vocal development follows an inborn program which is primarily determined by maturation of anatomical structures and neuro-physiological mechanisms underlying laryngeal and supra-laryngeal sound production^5–12^. The earliest vocal stages are commonly known as crying/phonation, primitive articulation (cooing) and expansion stage (pre-canonical babbling). Cooing describes a universal stage of vocal development at around 2-4-months of life. Cooing vocalisations may be of purely laryngeal origin (so-called vocants) or may include primitive supralaryngeal activity (articulation). According to Cruttenden^13^cooing is a kind of ‘intermediate stage’ between crying and babbling, although often the distinction is lost and it is subsumed under the more general term of babbling. So-called marginal babbling of 5-6-month-olds and the subsequent canonical babbling of 6-12-month-olds are vocalisations that are much more organised in terms of syllabicity than cooing^12^.

While the literature on canonical babbling in 6-12-month-olds is extensive, early vocal development in the first six months of life has received relatively scant attention^14,15^. Nonetheless, in the research which has been undertaken, studies have found striking similarities in features of pre-speech non-cry vocalisations by infants from different language groups^16–18^. In contrast, there has been increasing evidence for the shaping effects associated with ambient language. The existence of memory traces of prenatally perceived salient melodic (prosodic) features has been observed in perceptive studies, demonstrating newborns’ preference for listening to voices and languages that they experienced in the womb^19–23^. Moreover, the fundamental frequency contour (melody)^24–30^ and first consonant-like elements^16^ have been found to differ between newborns and infants from different cultures and their respective languages. A comparative study of cooing between healthy Cameroonian Nso and German infants aged 3 months revealed a high similarity of analysed sound features between both groups, pointing to relatively universal vocal development^16^. Notwithstanding this universality, one striking peculiarity in the vocal behaviour of the Nso infants was identified. Most (63%) Nso infants produced clicks in their cooing that resembled the typical para-linguistic clicks of adult Lamnso speakers^16^who are known for their affective usage of clicks^31^. As an inherent element of infant-directed speech, these clicks were frequently perceived by Nso infants, especially during dyadic face-to-face interaction.

Social interaction is opined to be an essential source of variability in vocal development during the first year of life^32,33^. Infants learn that their behaviour, particularly their vocal productions, directly affect their social environment. This likely explains the observed Nso infants’ clicks^16^. It provides a further example for the shaping influence of the ambient sound environment and underscores the position that early hearing matters in vocal development.

There is considerable evidence that the auditory experience of ambient language has effects on both infant speech production and speech perception in the first years of life^29,34–38^. Although, this viewpoint is not universally shared. In several studies of adult listeners’ perception of babbling in infants aged between 7 and 18 months from different language backgrounds^39–41^no detectable ambient language effects on infant ‘pure’ babbling was found. Only “utterances influenced by language-specific features of lexical items” were identified^41 [p.100]^. However, these findings do not prove that ‘pure’ babbling shows no language-specific elements, rather that adult listeners were unable to identify them in the performed listening experiments. There are various reasons why listeners might not be successful at identifying language-specific elements in infants’ babbling. Engstrand and colleagues suggested that “results of ambient language listening tests may depend crucially on judgments of vocalizations’ word status.”^39 [p.17]^ While, Canault and colleagues^42^ postulated that the effect of the language environmental input is also modulated by the degree of articulatory difficulty of the phonetic targets.

Taken together, results from the literature on the modifying effects of surrounding language on pre-speech sounds produced by infants may seem contradictory. However, we assert that this is not the case. Rather, the existing literature shows that the modulating effect of the surrounding language, and thus the significance of hearing in language development, is manifest when assessed based on acoustic properties and entities that are typical of the respective vocal developmental stage. Our study takes this into account by analysing the melodic and primitive articulative elements typical of the cooing stage. Our research question was: *Does a strongly reduced auditory feedback have a significant effect on laryngeal and/or supra-laryngeal activity in the production of the very first comfort sounds*,* namely during cooing*,* among infants aged 2–4 months?*

There is some evidence of the influence of auditory feedback on the vocal productions of typically developing infants aged from five months onwards, but there is relatively little research available among younger infants. For infants aged approximately 7 to 10 months, it was shown that the production of well-formed syllables (canonical babbling) that can function as the phonetic building blocks of later words were not observable in severely to profoundly deaf infants^43–45^. In hearing-impaired infants who have not yet received hearing aids, the onset of canonical babbling is delayed. Oller and colleagues found that nearly half (48%) of the variance in onset of canonical babbling was accounted for by age of amplification; the age when the infants first started using hearing aids^46^. Analyses of babbling of deaf and hearing infants have shown that auditory feedback is required to coordinate the movements of the phonatory and articulatory systems, and that this ability to coordinate is a prerequisite for the development of normal speech production^47^.

What about younger infants? Despite the non-negligible prevalence of congenital hearing impairment and the evidence for the importance of hearing at the earliest stages of vocal development, to the best of our knowledge there are only four studies that have investigated the vocalisations of hearing-impaired infants aged 2–4 months^47–50^. Cooing is easily elicitable in a face-to-face situation, and even deaf infants coo in such situations^47^. How the cooing of deaf infants differs from that of hearing infants has not been well studied. Some insight came from our preceding study of vocants (vowel-like vocalisations) in cooing of profound congenital sensorineural hearing loss infants aged 60–180 days^50^. Vocants, sometimes termed ‘quasivowels’^12^are one of the very first cooing sounds. They are produced laryngeally while the vocal tract remains in a neutral position; i.e., there is no articulation. Comparison of vocants produced by hearing impaired infants against normally hearing infants of the same age revealed significantly more complex melodies, especially double-arc structures, in normally hearing infants^50^. What is unknown is whether these findings generalise to all sounds beyond vocants found in the cooing stage. Such sounds include isolated closants, vocant-closant combinations, and resonance variations^51–53^ within the vocal tract that generate vowels.

Extending the vocant study, the aim for this study was to objectively analyse and compare the entire cooing repertoire of profoundly sensorineural hearing impaired and healthy infants. We sought to investigate laryngeal and supra-laryngeal sound production during cooing to establish a more comprehensive model of early vocal development in profoundly hearing-impaired infants and gain a better understanding of their apparent development prior to amplification or cochlear implantation. Unlike prior infant studies, this is the first quantitative investigation of a combined analysis of laryngeal activity (melodic structure) and supra-laryngeal activity (articulation) in individual cooing vocalisations at this early age. Based on Wermke and Mende’s *Melody-Development Model* (for details see^51,54,55^, we hypothesised that cooing vocalisations produced by infants with profound sensorineural hearing loss would show a delay in both, melody development and articulatory development compared to their age-matched healthy peers.

## Methods

### Study design

A case-control design was employed where melodic and articulatory features among infants aged between 2 and 4 months with profound sensory-neural hearing impairment (HI) were compared against an age-matched healthy young infant group with normal hearing (NH). The age range corresponds to the so-called primitive articulation stage^12,56^. At this age, intensive cooing (an early developing form of pre-canonical or marginal babbling) occurs usually in face-to-face interaction with caregivers.

### Participants

All infants were drawn from a larger cohort of moderate-to-severe HI and NH infants whose early language development has been longitudinally followed within an early research and intervention program. The prospective cohort includes all female and male infants (*n* = 128) born at the University Hospital of Würzburg between January 2018 and August 2019, who were referred to the Department of Otorhinolaryngology, University Hospital Würzburg because they failed their newborn-hearing-screening. Between the 10th and 24th week of life, vocalisations of these infants were recorded in addition to the routine second hearing test by auditory brainstem response (ABR). The control group consists of infants with normal hearing on this ABR test. Overall, 15 infants were eligible from this cohort (seven HI and eight NH infants). To enlarge the sample size, the analytical sample was augmented by eligible infants who had been the first patients/probands recruited within the program at the university hospital with the apposite archived recordings (*n* = 9, five HI and four NH infants). Inclusion from the retrospective cohort used the same audiological information and criteria.

Selection criteria were a full-term birth (although archived recordings included cooing of two late preterm HI infants, who were subsequently included to bolster the case sample size; see: Table S1 Supplementary information), pass (NH infants) or failure (HI infants) of the Brainstem Evoked Response Audiometry test, and no signs of a developmental disorder within the first 6 months of life. The psychomotor and cognitive development experienced by all the infants was normal: All infants received standardised medical examinations (called U-examinations). The first checkup occurred immediately after birth. At about 3–4 months, the fourth examination (U4) included an examination of the age-appropriate development and mobility of the infant, the organs, sensory organs, and an examination of growth, motor skills and the nervous system. Further, we selected those NH infants who had a cooing recording within their first 2–4 months of life and could be age-matched to the group of HI infants. Cranial magnetic resonance imaging examinations of those in the HI group revealed no anatomical abnormalities.

There were 12 (4 female, 33%) HI infants eligible from the cohort with profound congenital sensorineural hearing loss and 12 (5 female, 42%) age-matched NH infants selected as controls. Thus, in total, audio files of 24 German infants (with partially bilingual environment) at 70 to 127 days of life (mean 96.4 days) were analysed (see: Table S2 Supplementary information).

Among HI infants, hearing aids are routinely prescribed and tried out, even though their use is of little or no benefit in cases of congenital profound hearing loss. Four infants in the HI group received hearing aids within days of the first recording (range 1-7d); one infant (22-AF) received hearing aids 53d before the first sound recording (see Table 1). All infants in the HI group subsequently received cochlear implants (after our observation period). In order to confirm the final diagnosis of a profound hearing impairment and to initiate the appropriate treatment, some infants visited the Department of Otorhinolaryngology more than once during the observation period (*n* = 5 HI). Four NH infants were also repeatedly recorded (see: Table S2 Supplementary information). Each time, recordings were made according to the same protocol and within the identical setting.

**Table 1:** Audiological data for the infants of the HI group.

Subject	Click ABR	ASSR (0.5/1/2/4 kHz)	FF unaided (mean response over all frequencies)	Age [d] at first hearing aid fitting	Comments
BE	**r/l**: >100dB	**r/l**: 0.5 kHz: 90dB 1/ 2/ 4 kHz: 100dB	100dB	207	Genetic disorder (GJB2), most common gen for non-syndromic autosomal-recessive hearing loss
CQ	**r/l**: ?100dB	No measurement	95–100 dB	146	Familial deafness, communication in sign language
DA	**r/l**: >100dB	**r**: 0.5 kHz: 90dB 1 kHz: 100dB 2/4 kHz: >100 dB **l**: 0.5/1 kHz: >100dB 2 kHz: 95dB 4 kHz: 100dB	100dB	-	Severe bilateral cochlear malformation POU3F4 syndrome
DQ	**r/l**: >100dB	**r**: 0.5/1 kHz: 100dB 2/4 kHz: >100dB **l**: 0.5–4 kHz: >100 dB	100dB	163	
ER	**r/l**: >100dB	**r**: 0.5 kHz: 80dB 1/2 kHz: 85dB 4 kHz: 90 dB **l**: 0.5 kHz: 75dB 1/2 kHz: 85dB 4 kHz: 90 dB	> 100 dB	102	Auditory Neuropathy Spectrum Disorder (ANSD)
FF	**r/l**: >100dB	**r**: 0.5 kHz: 70dB 1 kHz: 90 dB, 2 kHz: 95dB 4 kHz: 90dB **l**: 0.5 kHz: 80dB 1 kHz: 85dB 2 kHz: 90dB 4 kHz: 95dB	0.5, 1 kHz: 85 dB 2 kHz: 95 dB 4 kHz: 75 dB	257	
FI	**r**: ?100 &90 dB **l**: >100 dB	**r**: 0.5 kHz: 100 dB 1 kHz: 95dB 2 kHz: 85dB 4 kHz: 90dB **l**: 0.5–4 kHz > 100 dB	> 90dB	104	Genetic disorder (GJB2), most common gen for non-syndromic autosomal-recessive hearing loss
**AF**	**r/l**: >100dB	**r/l**: 0.5 kHz: 80dB 1/2/4 kHz: >100dB	100dB	50	Familial hearing loss
AV	**r**: >100dB **l**: 100dB	**r/l**: 0.5/1 kHz: 95dB > 1 kHz: >100dB	100dB	89	Genetic disorder (GJB2), most common gen for non-syndromic autosomal-recessive hearing loss
AH	**r**: 90dB **l**: >100dB	No measurements	> 100 dB	77	
BO	**r/l**: >100 dB	**r/l**: 0.5 kHz: >80dB 1 kHz: 90dB > 1 kHz: >100dB	> 100 dB	153	Twin I, preterm birth Genetic disorder (LRTOMT), non-syndromic hearing loss
CO	**r/l**: >100dB	**r/l**: 0.5/1/2/4kHz: >100dB	> 100dB	153	Twin II, preterm birth Genetic disorder (LRTOMT), non-syndromic hearing loss

ABR, auditory brainstem response; ASSR, auditory steady state-response; FF, free field; r, right ear; l, left ear; or “?”, questionable response.

### Procedure

Recordings were made in the Department of Otorhinolaryngology of the University Hospital Wuerzburg following a standardized hearing test using ABR or, in the case of repeated measurements, preceding a medical consultation. This first hearing test was carried out as standard between the ages of 70 and 115 days of life. Further clinical appointments were not standardized but individually determined. A series of sound recordings were made during this period. The aim of the study was to record cooing vocalisations in a communicative situation (face-to-face interaction) between 70 and 130 days of age. The timing of the recordings (see Table S2 in the Supplementary information) varied between infants and could not be better standardised, as clinical requirements and family circumstances always took precedence. This is the reason why for some infant recordings were only available at individual points in time, while for other infant recordings were available at several points in time during the observation interval (see Table S2 Supplementary information). All available records were utilised.

Infants were placed directly in front of and facing their mother. Mothers were instructed to interact with their infant just as she would do at home when she had a few minutes to spend with the baby. Mothers were allowed to talk to their infants, but always to fell silent when the infant started to vocalize. An examiner, present in the background, switched on the recording device. Digital (45 kHz Fs, 16 Bit) recordings of infants’ comfort vocalisations were made using a TASCAM DAT recorder (DR-100) equipped with an external Earthworks microphone (TC20). The recording took place within a sound booth or noise-reduced room, with the distance between the microphone and the infant’s mouth being approximately 15 cm. All the comfort vocalisations an infant produced were recorded. Recordings were generally stopped after 20 min, but earlier in the case of fussing or crying by the baby.

### Ethics

The study was approved by the medical ethical board of the University Wuerzburg (#308/17) and was carried out in accordance with relevant guidelines and regulations; informed consent signed by parents was given. All infants were participants of an early research and intervention program. As described above, the dataset of the present study was enlarged by including archived recordings of five HI and four NH infants who had been the first patients/probands recruited within the program at the university hospital. Their data were obtained based on the same protocol and ethical approval was provided by the same board of the University Hospital Wuerzburg (# 143/04). All recordings and analyses were archived as anonymized datasets.

### Pre-processing of recordings

**Fig. 1:**
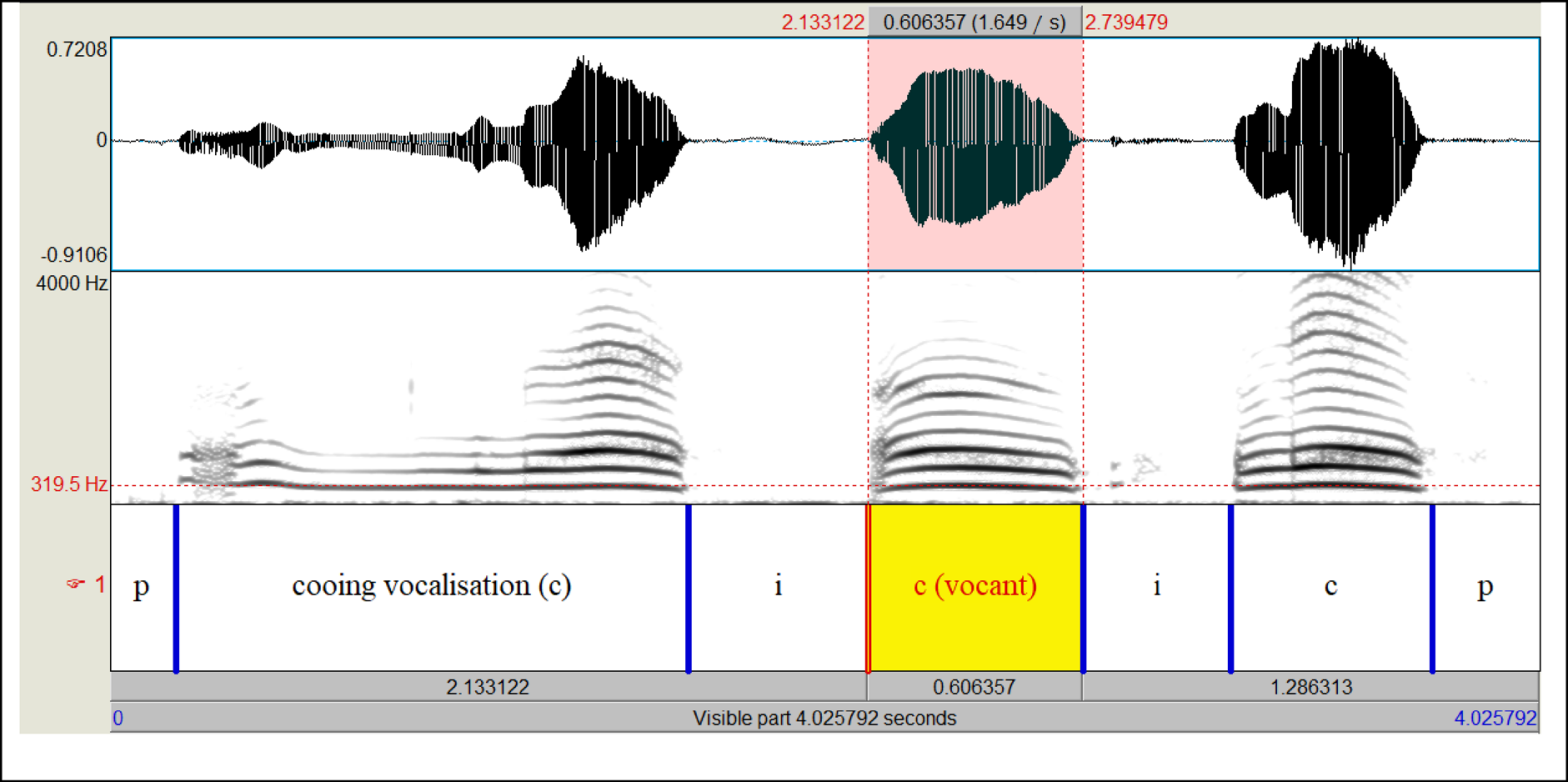
Example of an annotated cooing sequence in PRAAT. The upper section of the PRAAT output window displays the amplitude of the recorded sequence. The middle section shows the frequency spectrogram (frequency range linear 0–4 kHz). The lower area of the output window shows the annotation variables of the individual events. Here, the sequence consists of pauses (p) and three cooing vocalisations (c) separated by inspiratory intervals (i). Unlike the other two coos, the cooing vocalisation marked in yellow contains no supra-laryngeal activity (vocant).

Pre-processing was performed to identify single cooing vocalisations within the recorded audio files (*.wav). Using a script^57^ of the open-source software PRAAT v. 6.0.40^58^, it is possible to mark single acoustic events encompassing all types of egressive and ingressive vocalisations (produced when breathing out or in) of the infant, silent intervals, speech, and background noise by interval cursors (vertical lines in Fig. 1). The marked time intervals are labelled below the frequency spectrograms, a process called annotation. In Fig. 1, we displayed an example of a PRAAT output window showing the result of an annotation process of a cutout of a recorded audio sequence of 4s duration. The displayed sequence starts with a Pause (p), which is followed by three cooing vocalisations (c) which are each separated by inspiratory breaks (i). The sequence is terminated by a further Pause. A cooing vocalisation was defined as the onset and the offset of identifiable acoustic energy in the waveform (amplitude) that occurred on the expiratory phase of a single respiratory cycle (upper section in Fig. 1). To identify cooing vocalisations within the original audio file (*.wav format), we used an automatic PRAAT routine for this segmentation process^57^. All automatic segmentations were labelled and double-checked by an audio-visual analysis by two of the authors (SA, KW). Identified segmentation errors by the detection algorithm, which sometimes occurred in cases of low sound intensity, were manually corrected by changing the cursor positions for the detection of the start and end points of events. This process was also carried out independently by SA and KW for all vocalisations included within the study. A high level of agreement (99%) was achieved. The discordant cases (1%) were subsequently reidentified and jointly reconsidered, and a consensus decision made. Among all annotated events, the time intervals comprising cooing vocalisations were identified by an audio-visual analysis using frequency spectrograms and time representations (waveform), manually annotated, and then automatically saved.

### Cooing vocalisation analysis

In a next step, all cooing vocalisations were analysed with regard to their acoustic characteristics. Prelinguistic development is a continuous and dynamic process. As part of this process, the infant experiments with different parts of the sound system during this time. In principle, there are two main levels in which the individual components are tested independently and in interaction with each other: the laryngeal and the supra-laryngeal level. On both levels, sound production is influenced by auditory feedback and therefore hearing. At the laryngeal level, variation includes the pitch (fundamental frequency), intensity, duration, and melodic complexity. To determine variation at the laryngeal level, we analysed the fundamental frequency contour (melody) to differentiate between simple and complex melodic structures (see Analysis of Laryngeal Activity). At the supra-laryngeal level, the formation of articulatory elements in cooing involves muscular activity at different anatomical structures such as the pharynx, velum, nose, palate, jaw, or lips which can be stimulated individually or in combination. It is often unclear what exactly happens at which locations in the vocal tract during cooing. Therefore, for each cooing vocalisation, we only determined whether it contained any kind of articulatory activity or not; i.e., we distinguished between cooing vocalisations with and without articulation. We believe that this is one of the best ways to objectively assess prelinguistic skills at this early age, given the considerable inter-individual developmental variability and immaturity of articulation (see Analysis of Articulatory Activity). Each cooing vocalisation was thus evaluated on two levels: (1) in terms of its melodic structure (simple vs. complex); and (2) in terms of whether or not it contained any form of articulatory activity.

### Analysis of laryngeal activity (Melody)

**Fig. 2:**
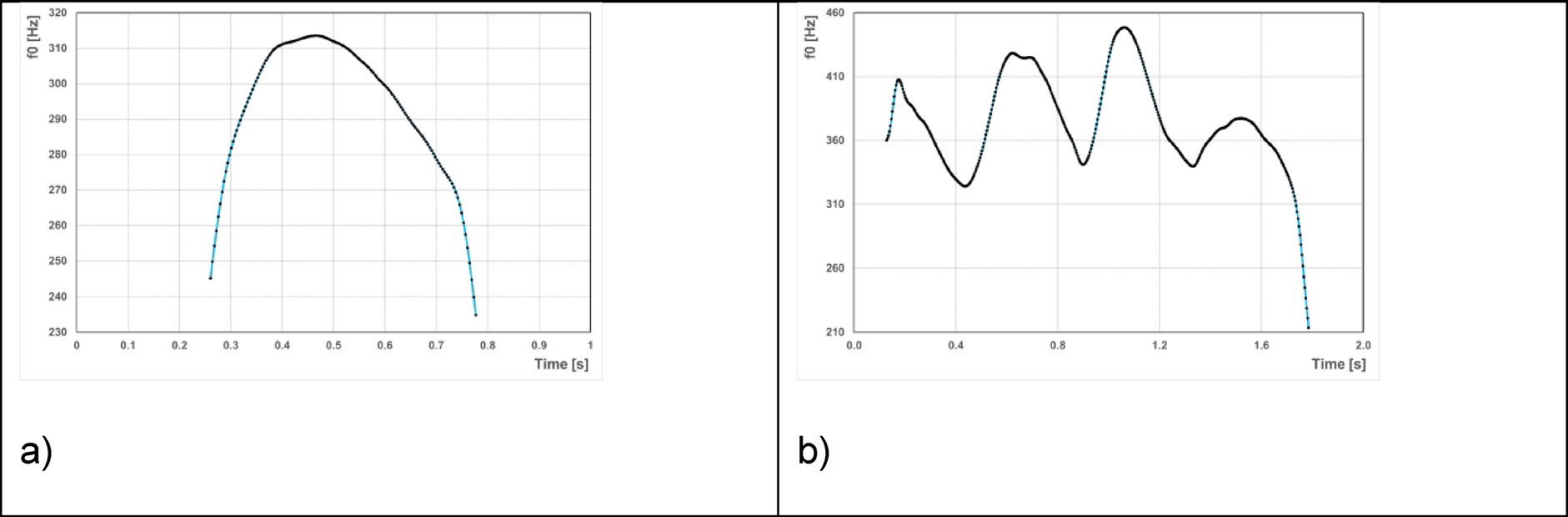
Melody diagrams (time-fundamental frequency representations) exemplifying cooing vocalisations with (**a**) a simple, i.e., one-arc melody, and (**b**) a complex, multiple-arc melody.

As described in detail elsewhere^54^for each cooing vocalisation the fundamental frequency (f_0_) was automatically analysed using PRAAT and thereafter transferred to a further software system. Melody analysis was performed using specific in-lab software (CDAP, pw-project), which was implemented as a routine procedure at the Center for Pre-Speech Development and Developmental Disorders. Using the f_0_ data calculated with PRAAT, the CDAP software allows for flexible drawings of melody diagrams and quantitative melody structure analysis^54^. Based on objective criteria (for details, see^54^, all cry melodies were analysed and subdivided into those with only a simple (single-arc) melody (Fig. 2a) and those with a complex (multiple-arc) melody (Fig. 2b). This avoided subjectivity in the coding of melodic structure. The classification into cooing vocalisations with or without a complex melody (binary 0/1 variable) was quantitatively performed using fundamental frequency-time diagrams. A complex melodic structure exhibits ≥ two melodic arcs and/or inner-melodic pauses between arcs by glottal oscillatory pauses or marked laryngeal constrictions^59^ that generate rhythmical variations of the acoustic gestalt^54,55,60–63^. Figure 2 displays examples for cooing vocalisations with a simple and a complex melody, respectively. This classification is based solely on activity at the laryngeal level and is independent of whether a cooing vocalisation demonstrates activity at the supra-laryngeal level; i.e., whether it contains an articulation or not.

### Analysis of articulatory activity

While the analysis of laryngeal activity was aimed at characterising the melodic structure of infant cooing, the analysis of articulatory activity was aimed at characterizing sound features produced in the vocal tract. The task is complex in that the supra-laryngeal system is still in a maturing and growing state in terms of the anatomical structures and neurophysiological control mechanisms involved^8^. As a result, cooing lacks well-formed vowels with their speech-like formant characteristics, clearly articulated consonants, and mature syllables as they are typical for speech^12^. Nevertheless, the articulatory activity observed in cooing is diverse, even if it is specific, and results in very different acoustic sound phenomena. For example, articulatory activity was observed in the form of raspberries, squeals, growls, pharyngeal/velar vibrations, nasal consonants and first primitive syllables during the observation age. Consonant-like sounds (closants)^64^ and their combinations with vowel-like sounds (vocants)^64^ were typical. Consonant-like articulations were predominantly produced in the back of the vocal tract. Moreover, the articulatory activity included full vowels which cause the auditory impression that the vocal tract is postured, with intentional positioning of the mouth and tongue in a speech-like way^12^. Full vowels differ from vocants in that their vowel quality is distinct from that corresponding to an at rest (neutral) position of the vocal tract. Cooing vocalisations are produced with a neutral vocal tract when none of the articulators (e.g., jaw, tongue, velum) are moving. The cooing vocalisations produced in this way are solely the result of phonation (laryngeal activity) that is not accompanied by differentiated articulatory activity^65^.

**Fig. 3:**
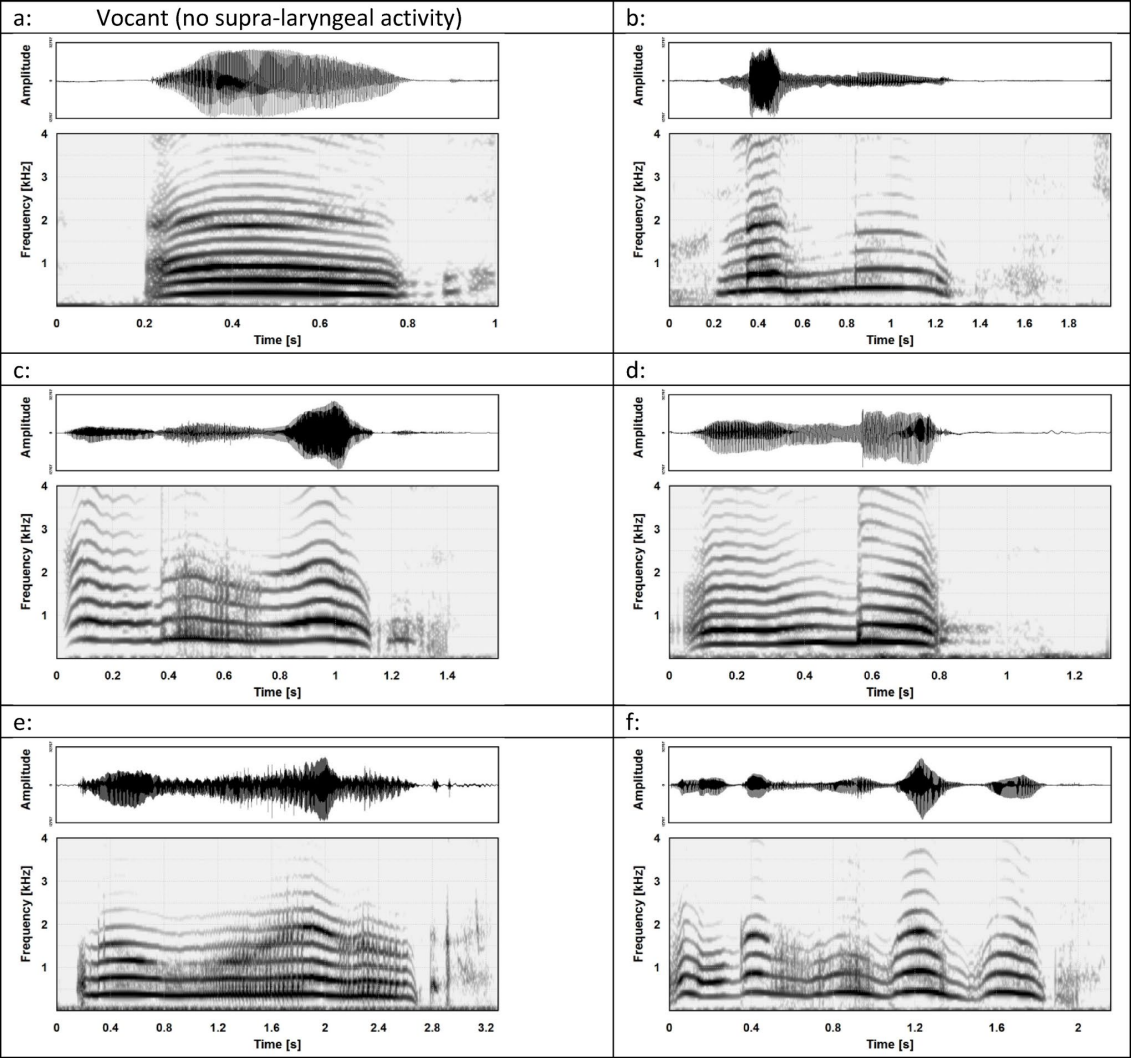
Frequency spectra (linear, up to 4 kHz; time representation above) of individual cooing vocalisations. Note that all examples also display the inspiratory noise following the coo. Example (a) displays a vocant (vowel-like vocalisation or ‘quasivowel’), while the other examples show coos with supra-laryngeal activity, well recognisable by the occurrence of consonant-like elements (vertical structures). For the original audio files of these vocalisations see Supplementary Information.

The analysis of articulatory activity was simplified to take account of the complexity and characteristics of an immature system under development. All cooing vocalisations were assessed auditorily and visually, using the corresponding frequency spectrograms, to determine whether or not they contained any kind of articulatory activity (binary 0/1 variable). Any form of articulatory activity in cooing can be heard very well. In addition, the frequency spectrograms help to recognise the articulatory elements. They are characterised by short noise-like bands or vertical lines. This can be clearly seen in the examples in Fig. 3. Full vowels can be recognised by variations in the intensity distribution on the harmonics of the frequency spectrum. Beside visual identification, these intensity variations are also very easy to identify by ear (for details see^50–52^. The identification of articulatory activity was performed by one of the authors (SA). Typical examples of cooing vocalisations without and with articulatory activity are shown in Fig. 3. The corresponding sound files are provided in Supplementary information.

### Statistical analysis

The STrengthening the Reporting of OBservational studies in Epidemiology (STROBE) guidelines informed reporting of the study findings^66^. Initially, the participants and their vocalisations were described. Age between HI and NH groups was assessed using Student’s t-test. Next, the distribution of melody structure and articulatory activity for the vocalisation was presented, together with their distribution of concordance. Inter-rater reliability of the coding procedure was tested on a randomly selected sub-sample of 150 cooing vocalisations and assessed using Cohen’s κ. Multilevel mixed-effect modified Poisson regression models (with log-link function and robust variance estimators) were employed to analyse vocalisations. The multilevel mixed-effect structure was chosen as vocalisations were nested within infants, and infants could be modelled as random intercepts. Moreover, given the likely intra-infant correlation between their vocalisations, an exchangeable covariance structure was employed, and infant age at vocalization measurement was included in all models. The modified Poisson likelihood function was selected as melody complexity and articulation are not rare events^67^. Prevalence ratios (PRs) and associated 95% confidence intervals (CIs) between HI and NH groups were reported from these models. Finally, as one child (#10-AD) with normal hearing appeared to have relatively higher rates of articulation, a sensitivity analysis was conducted removing that individual and re-running the final model – to ascertain their potential influence on the derived estimates. All analyses were conducted using Stata SE version 18.0 (StataCorp, College Station, USA), and a two-tailed α = 0.05 denoted significance.

## Results

### Participants’ vocalisations

The available database included 2,463 cooing vocalisations from 24 infants (*n* = 12 NH infants: 1,247 vocalisations; *n* = 12 HI infants: 1,216 vocalisations). However, 56 (4.6%) NH and 87 (7.0%) HI infants’ vocalisations were excluded due to crying or fussing vocalisation contamination, leaving 2,320 cooing vocalisations (*n* = 1,160 in each group). Overall, there were a median of 55.5 vocalisations per infant (range: 14–236). The mean age at first recording was 96.9 days (SD = 14.6) for NH infants and 95.8 days (SD = 12.4) for HI infants, a difference that was not significant (*p* = 0.85). Table S2 in the Supplementary information provides additional features of the dataset. Inter-rater reliability of the coding procedure for cooing vocalisations with and without articulatory activity between SA and KW was found to be κ = 0.92, representing a high level of agreement.

### Descriptive analysis of melodic structure and articulatory activity

Table 2 presents the distribution of melodic structure and articulatory activity detected in the vocalisations made by infants within the HI and NH groups. Clear differences emerged, with NH infants’ vocalisations more like to have melodic complexity and articulations.

**Table 2:** Distribution of melodic structure and articulatory activity **(ART)** in vocalisations by hearing impaired and normal hearing groups.

		Hearing impaired (HI)		Normal hearing (NH)		Total	
		n	(%)	n	(%)	n	(%)
Melodic structure (laryngeal activity with or without ART)							
Simple		760	(65.5)	460	(39.7)	1,220	(52.6)
Complex		400	(34.5)	700	(60.3)	1,100	(47.4)
Articulatory activity (with either simple or complex melody)							
No		830	(71.6)	546	(47.1)	1,376	(59.3)
Yes		330	(28.4)	614	(52.9)	944	(40.7)
Melodic structure and articulatory activity							
Otherwise		970	(83.6)	684	(59.0)	1,654	(71.3)
Both*		190	(16.4)	476	(41.0)	666	(28.7)

Note: *Both defined as having complex melodic structure and supra-laryngeal activity (articulation). Otherwise: vocalisations with single-arc melody /no ART, single arc melody /ART or complex (multiple arc) melody/ no ART.

Among the NH infant vocalisations, 476 (41.0%) had both a multiple-arc (complex) melody combined with articulatory activity (ART). Otherwise, vocalisations were observed that contained either a simple (single-arc) melody (*n* = 322, 27.8%) or complex melody (*n* = 224, 19.3%), but exhibited no ART. Lastly, 138 (11.9%) vocalisations contained a simple melody and ART. However, among HI infant vocalisations: 190 (16.4%) exhibited complex melody and ART; 620 (53.4%) had simple melody; 210 (18.1%) had complex melody and no ART; and 140 (12.1%) vocalisations had a simple melody with ART.

### Multilevel mixed effects models comparing groups

Multilevel mixed effects modified Poisson regression models were employed, with vocalisation nested within infants, and infants treated as random intercept effects. Table 3 gives the estimated PRs and associated 95% CIs of melodic complexity and supra-laryngeal articulations in vocalisations for NH infants compared to their HI counterparts derived from these models, adjusted for age. In the model investigating melodic structure, the estimated PR for complex structure among NH infants was 1.56 (95% CI: 1.12, 2.18) higher than that of HI infants. Infant age was not statistically related to changing melodic structure prevalence (*p* = 0.73). The infants’ random effects term was significant, with the intercept variability given by SD = 0.360 (95% CI: 0.227, 0.571).

**Table 3:** Multilevel mixed effects modified Poisson regression models estimating the prevalence ratios (PRs) and associated 95% confidence intervals (CIs) of melodic complexity (MC) and articulatory activity (ART) in vocalisations for normal hearing infants compared to their hearing impaired counterparts, after adjusted for age.

Multilevel mixed-effects model	PR	(95% CI)
*Melodic structure regression model*		
Melodic complexity		
Simple	1	(reference)
Complex	1.56	(1.12, 2.18)
Age (days)	1.002	(0.992, 1.011)
*Articulatory activity regression model*		
Articulatory activity		
No	1	(reference)
Supra-laryngeal activity (ART)	1.91	(1.11, 3.27)
Age (days)	1.011	(1.005, 1.011)
*Melodic structure and articulatory activity regression model*		
Melodic complexity and articulatory activity		
Otherwise	1	(reference)
Both*	2.33	(1.34, 4.07)
Age (days)	1.015	(1.009, 1.020)

*Both defined as having complex MC and supra-laryngeal articulation ART. Otherwise: vocalisations with single-arc melody /no ART, single arc melody /ART or multiple arc melody/ no ART.

**Fig. 4:**
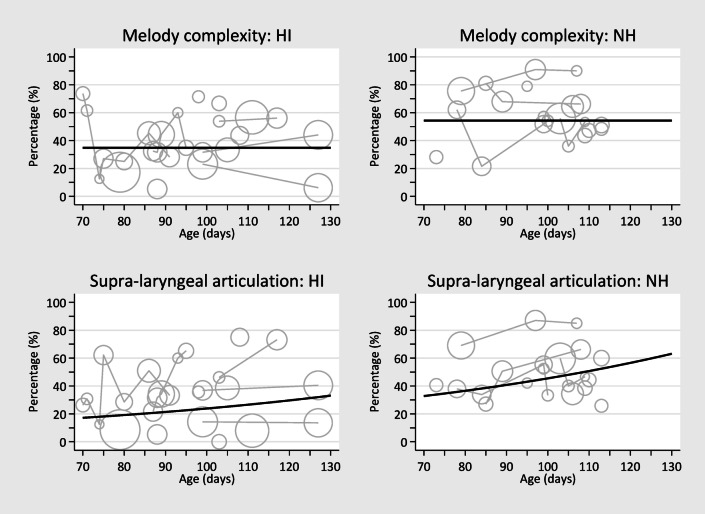
Bubble plot of percentage of melody complexity and articulation in infants’ vocalisations with connections between bubbles for those measured over multiple days, together with the superimposed estimated group mean (solid black line), stratified by hearing groups (note: the bubble size represents the relative sample size between infants).

A bubble plot of the melody complexity percentage in infant’s vocalisations with connections between bubbles for those measured over multiple days, together with the superimposed estimated group mean (solid black line), is depicted in Fig. 4. The estimated melody complexity percentage mean among HI and NH infants was 34.8% and 54.3%, respectively.

For the vocalisations with ART and either a simple or complex melody, the estimated PR was 1.91 (95% CI: 1.11, 3.27). In this analysis, ART significantly increased with age – as also depicted in Fig. 4. A check was undertaken to identify whether this increase in age may be differential between NH and HI groups, however no significant interaction was found (*p* = 0.65). The estimated mean proportion of ART in vocalisations among the hearing impaired was 17.2% at 70 days increasing to 33.1% at 130 days, while the estimated mean proportion among those with normal hearing was 32.8% at 70 days increasing to 63.1% at 130 days. In this model, the infants’ random effects term was also significant, with variability given by SD = 0.564 (95% CI: 0.398, 0.800).

When investigating the combined melodic structure and ART, the measured PR was 2.33 (95% CI: 1.34, 4.07). Again, these rates increased with increasing age (see Table 3) and, again, no age×group interaction was observed (*p* = 0.78). The estimated mean proportion of complex melodic structure and ART in vocalisations among the hearing impaired was 9.2% at 70 days increasing to 22.2% at 130 days, while the estimated mean proportion among those with normal hearing was 21.4% at 70 days increasing to 51.7% at 130 days. The infants’ random effects term was again significant in this model, with variability given by SD = 0.564 (95% CI: 0.396, 0.803).

### Sensitivity analysis

The combined melodic structure and ART vocalisation model was re-run, excluding the infant (#10-AD) with normal hearing who appeared to have relatively higher rates of ART (see Fig. 4). The significant group difference remained (*p* = 0.007), as did the age effect (*p* = 0.001). Naturally, the estimated effect size was dampened (PR = 2.08; 95% CI: 1.22, 3.56). This demonstrates that the difference between groups was not contingent on this one infant.

## Discussion

This study demonstrated the impact of a profound limitation of auditory feedback by sensory-neural hearing loss on infant cooing. A significant lower melodic complexity, i.e., laryngeal flexibility, and less frequent articulatory activity were found among cooing vocalisations of the hearing-impaired infants compared to their normal hearing peers. We found that the HI group produced significantly fewer cooing vocalisations that contained both a complex melody and elements produced by articulatory activity in the vocal tract. A key finding and strength of the study is the age-dependent development of the acoustic properties analysed, which was almost identical in both groups. What we saw was a universal developmental pattern; an increasing ability to produce complex melodic structures with the vocal folds and then combine these with simple articulatory movements in the vocal tract. One plausible explanation is that of maturational gradients in functional developmental modules (DFMs), introduced by Kent^65^. For the current developmental stage, the emergence of complex structures in both infant groups is primarily explained by the Pharyngo-Laryngeal DFM in interaction with the Laryngeal DFM^8^.

Vocal development can best be described as the addition of upper pharyngeal and oral modulations to an already well-developed laryngeal vocal coordination. This development appeared to be delayed in HI infants. This lends support to the idea of an innate program for melody development, one that is receptive to learning from both self-generated and ambient sounds^51,54,55^. Well-functioning auditory feedback is necessary for the acquisition of melodic variations as essential component of prosody acquisition of the surrounding language.

By 12 weeks of age, infants begin to imitate vowels presented to them^68^. This suggests that infants experience within the first weeks of life that certain articulatory movements have certain auditory consequences, leading to the development of a link between perception and motor movement of the vocal tract. Although the neurophysiological basis of this link is not yet fully understood, there are recent theories that addressed the convergence of the auditory system and the motor system^69,70^.

A widely varied repertoire of different articulative elements was reported for the investigated age period of approximately 2.5-4 months^47,71^. The occurrence of many different elements supports the hypothesis that infants begin to experiment with the possibilities of their vocal tract and play with different articulators during the age period studied. However, the anatomy of the Pharyngo-Laryngeal DFM still constrains actions of the articulators but also allows for infant-specific articulatory capabilities^8^.

Our findings suggest that the primary developmental challenge in vocal development lies not exclusively in the geometry and acoustics of the vocal tract, but with the maturation of neurophysiological control systems that coordinate phonatory and articulatory functions^55,65^. These systems include different sensory feedback types, particularly auditory and visual.

The time function of the fundamental frequency (melody) is a key quantity for characterizing infants’ utterances during the first months of life. From a physiological point of view, laryngeal phonation and vocal tract-based articulation are anatomically different and independently controlled systems. For speech acquisition these two systems must interact systematically. The human infant needs to continually modify their laryngeally produced melodies and tune them to the resonant frequencies of a vocal tract that is constantly growing and changing. Most vocal tract structures appear to grow continuously from two weeks to about six years of age, with somewhat faster growth during the first 18 months of life^72^. The voluntary mastering of interaction phenomena between vocal folds and vocal tract structures is an essential prerequisite for performing fast and accurate shifts between vowel formants in cooing, babbling and later speech^51^. Neurophysiological fitness of the underlying control systems, including auditory feedback, are indispensable for this development.

In summary, these results suggest a unidirectional development towards language which, for all its individual variation, is far from being a randomly generated pattern. The fact that the first comfort vocalisations (cooing) can be triggered, particularly in face-to-face situations, and that these situations trigger an almost compulsive impulse to vocalise, suggests that these developmental patterns have deep evolutionary roots. The characteristics of the described vocalisations uttered within a face-to-face proto-conversation, provide a new perspective on early mechanisms involved in learning to talk.

This study has both strengths and weaknesses. Its prospective collection of robust data and careful analysis is a salient strength. While our sample size appears relatively small, it is the largest to date in infants this age and captured more than 2,300 vocalisations. Moreover, it was sufficiently large to demonstrate important clinical and statistical differences. However, the small number of cases limits conceptual generalization. To characterize the developmental path in more detail, it would be necessary to better capture individual variations in longitudinal studies with more frequent sampling. A phonetic analysis of the articulative elements and their position within the melodic contour might also help to better understand the universal mechanisms at work in cooing and to separate them from simple maturation phenomena. Just as the position of consonants in words is an important factor in their phonetic structure, readability and meaning, the position of articulative elements (consonantal precursors) could alter how sounds are perceived by social partners. This, in turn, could change their speech. Changes to the linguistic structure of adult speech in response to infant vocalisations may guide the earliest stages of language acquisition. This phenomenon was observed in a cross-linguistic study of 13 languages from five language families (within infants aged 5–30 months)^33^. The authors found that most caregivers significantly simplified their linguistic structure when responding to children’s immature speech. Experience gained from hearing oneself and others paves the way of vocal development towards language^73^.

Additionally, different positions of articulative elements within the melodic contour could be viewed as preliminary exercises for the placement of consonants within syllable structures at later ages. Recording these exercises could provide further objective data to help answer questions of language-specific influences on infant pre-speech sounds. Moreover, collection of a broader array of potential confounders would also be useful for future research (e.g., motor development, parent attachment, musical environment).

Knowledge of the described early vocal mechanisms is extremely limited^15^. Despite the benefits of newborn hearing screening (NHS), which is now practised in many countries, it often takes weeks to months before a definitive diagnosis of a congenital hearing impairment requiring treatment can be made. Supplementing perceptual screening (e.g., auditory brainstem response) with production screening (vocalisation analyses) may be a promising easy-to-implement tool to obtain additional information – especially in the era of artificial intelligence. This could lead to a more rapid diagnosis and the associated initiation of therapy. In many countries, routine NHS forms part of the standard neonatological assessment. Several reports suggest that these screening methods are highly reliable. However, there are reports of ≥ 2.5% of false-positive rates in NHS that require a multi-stage strategy which places not only greater demands on the limited resources of the healthcare system but causes a time delay in final diagnosis of inborn hearing disorders^74^. One promising strategy for reducing the initial NHS’s false-positive rates may be to include the analysis of spontaneous neonatal crying or early non-cry vocalisations in the pedaudiological diagnosis. Future studies, with sufficiently large case numbers, could ascertain whether this leads to a reliable reduction in false-positive rates. This might also help to establish earlier more individualised treatment. Moreover, improving the efficiency of screening programmes could indirectly improve communication and vocal development. It reduces the emotional strain on parents of neonates who belong to the false-positive group, which may affect their behaviour towards their infants^74^. Vocal analysis also provides an opportunity to objectively assess the individual benefit of hearing aids for young infants and to help optimise hearing aid fitting. From both a developmental and clinical perspective, it is important to continue this type of research into the first steps of human infants on their path toward language. This will offer a manifold of reliable comparisons to other ‘vocal production learners’ among talented non-human vocalists.

## Supplementary Information

Below is the link to the electronic supplementary material.

Supplementary Material 1

Supplementary Material 2

Supplementary Material 3

Supplementary Material 4

Supplementary Material 5

Supplementary Material 6

Supplementary Material 7

## Data Availability

Because the participants did not give explicit written consent that their data can be made publicly available, data will not be shared. The original dataset presented in the study is included in the Supplementary Material: further inquiries can be directed to the corresponding author.
