# Supplementary material for: Melodic and articulatory development is delayed in deaf infants aged 2–4 months
Source: Sci Rep. 2025 Aug 26;15:31357. doi: 10.1038/s41598-025-16820-w (PMC12381039; doi:10.1038/s41598-025-16820-w)
Supplement: Supplementary file 7 — Supplementary Material 7 [file 41598_2025_16820_MOESM7_ESM.docx]

**Supplementary materials for:**

**Melodic and articulatory development is delayed in deaf infants aged 2-4 months.**

- Table S1: Subject characteristics
- Table S2: Age distribution for individual recordings
- *Sound examples*

**Table S1:** Subject characteristics.

| **Group** | **Subject** | **Sex** | **GW**  **[weeks + days]** | **BW [g]** | **BL [cm]** |
| --- | --- | --- | --- | --- | --- |
| NH | 1-BJ | F | 39+5 | 3195 | 50 |
|  | 2-BX | M | 41+0 | 3140 | 51 |
|  | 3-DI | M | 40+4 | 3090 | 50 |
|  | 4-DL | M | 38+3 | 3350 | 49 |
|  | 5-DT | M | 41+0 | 3412 | 54 |
|  | 6-EG | M | 40+4 | 3650 | 57 |
|  | 7-FG | F | 38+4 | 3590 | 50 |
|  | 8-AA | M | mv | mv | mv |
|  | 9-AC | M | 38+0 | 4095 | 53 |
|  | 10-AD | F | 40+0 | 3790 | 55 |
|  | 11-AK | F | mv | mv | mv |
|  | 12-AD | F | 41+0 | 3120 | 51 |
| HI | 13-BE | F | 41+3 | 3960 | 51 |
|  | 14-CQ | M | 39+4 | 4455 | 56 |
|  | 15-DA | M | 41+2 | 3810 | 50 |
|  | 16-DQ | M | 39+2 | 4060 | 52 |
|  | 17-ER | M | 38+4 | 3745 | 54 |
|  | 18-FF | M | 40+2 | 3255 | 50 |
|  | 19-FI | F | 40+6 | 3540 | 48 |
|  | 20-BM | F | 34+5 | 2315 | 46 |
|  | 21-CM | F | 34+5 | 2310 | 47 |
|  | 22-AF | M | 40+0 | 3420 | 53 |
|  | 23-AH | M | 40+0 | 3945 | 55 |
|  | 24-AV | M | 37+3 | 3630 | 53 |

Note: BW: birth weight; BL: birth length; HI: hearing-impaired group; NH: normally hearing group; m: male; mv: missing value; GW: week of gestation; f: female; subjects 20-BM and 21-CM were twins.

**Table S2:** Age distribution for individual recordings and number of analysed sounds.

| **Group** | **Subject** | **Age[d] and (number of cooing vocalisations)** | | | | | |
| --- | --- | --- | --- | --- | --- | --- | --- |
|  |  | **70-79** | **80-89** | **90-99** | **100-109** | **110-119** | **120-129** |
| NH | 1-BJ |  |  |  |  | 113 (31) |  |
|  | 2-BX |  |  |  | 106 (90) |  |  |
|  | 3-DI |  |  | 99 (21) |  |  |  |
|  | 4-DL | 73 (32) |  |  |  |  |  |
|  | 5-DT |  |  |  |  | 113 (45) |  |
|  | 6-EG |  |  |  |  | 110 (36) |  |
|  | 7-FG |  |  | 95 (19) |  |  |  |
|  | 8-AA |  |  |  | 103 (179) 105 (25) 109 (15) |  |  |
|  | 9-AC | 78 (58) | 84 (65) | 99 (56) | 100 (24) |  |  |
|  | 10-AD | 79 (139) |  | 97 (77) | 107 (20) |  |  |
|  | 11-AK |  | 85 (37) 89 (81) |  | 108 (71) |  |  |
|  | 12-AD |  |  |  | 109 (39) |  |  |
| HI | 13-BE |  | 88 (38) |  |  |  |  |
|  | 14-CQ |  |  |  |  | 111 (113) |  |
|  | 15-DA | 79 (162) |  |  |  |  |  |
|  | 16-DQ |  |  |  | 108 (32) |  |  |
|  | 17-ER |  |  |  | 103 (21) |  |  |
|  | 18-FF |  |  | 98 (14) |  |  |  |
|  | 19-FI |  |  |  | 105 (57) |  |  |
|  | 20-BM |  |  | 99 (91) |  |  | 127 (81) |
|  | 21-CM |  |  | 99 (38) |  |  | 127 (84) |
|  | 22-AF |  |  |  | 103 (13) | 117 (41) |  |
|  | 23-AH | 70 (19) 71 (13) 74 (8) 75 (37) | 80 (28) 86 (53) | 91 (39) |  |  |  |
|  | 24-AV |  | 87 (37) 88 (38) 89 (70) | 93 (10)  95 (23) |  |  |  |

Explanation: For individual infants, age at each recording is indicated and the respective number of sounds in brackets. Three recordings of the infant #23 took place while the infant wore his hearing aids. Three other infants (#17, 19, 24) had already been fitted with hearing aids during the study period. However, they did not wear their hearing devices during the recordings.

**Sound examples corresponding to Fig. 3**:

- File_Figure 3a.wav
- File_Figure 3b.wav
- File_Figure 3c.wav
- File_Figure 3d.wav
- File_Figure 3e.wav
- File_Figure 3f.wav
